# 5-Year Clinical and Radiographic Results of the Direct Anterior Approach for Total Hip Arthroplasty Using a Collared Cementless Femoral Short-Stem Prosthesis

**DOI:** 10.3390/jcm11020346

**Published:** 2022-01-11

**Authors:** Ali Darwich, Kim Pankert, Andreas Ottersbach, Marcel Betsch, Sascha Gravius, Mohamad Bdeir

**Affiliations:** 1 Department of Orthopaedic and Trauma Surgery, University Medical Centre Mannheim, Medical Faculty Mannheim, University of Heidelberg, Theodor-Kutzer-Ufer 1-3, 68167 Mannheim, Germany; marcel.betsch@umm.de (M.B.); sascha.gravius@umm.de (S.G.); bdeir.m@hotmail.com (M.B.); 2 Department of Knee Surgery, Schulthess Clinic, Lengghalde 2, 8008 Zurich, Switzerland; Kim.Pankert@kws.ch; 3 Department of Orthopaedics, Hospital Centre Oberwallis, Ueberlandstrasse 14, 3900 Brig, Switzerland; andreas.otterbach@hopitalvs.ch

**Keywords:** cementless, collared, short stem, total hip arthroplasty, radiolucencies, HOOS

## Abstract

The aim of this study was to investigate the radiological and clinical outcome of the direct anterior approach (DAA) in total hip arthroplasty (THA) using a collared cementless femoral short-stem. This retrospective study included 124 patients with 135 THAs operated from 2014 to 2016 using a collared cementless triple tapered hydroxyapatite-coated femoral short-stem (AMIStem H Collared^®^, Medacta International, Castel San Pietro, Switzerland) implanted with a DAA. Follow-up was performed at three months, 12 months, and five years. Clinical outcome was assessed using the hip osteoarthritis outcome score (HOOS) and radiological analysis was done using conventional radiographs, which included evaluation of the femur morphology based on Dorr classification, of radiolucencies based on the Gruen zone classification and of stem subsidence. The mean age was 67.7 ± 11.3 years and the mean body mass index (BMI) was 27.4 ± 4.4 kg/m^2^. The stem survival rate at five years was 99.1% with one revision due to recurrent dislocations. Mean HOOS score improved from 40.9 ± 18.3 preoperatively to 81.5 ± 19.7 at three months, 89.3 ± 10.9 at 12 months, and 89.0 ± 14.0 at five years (all with *p* < 0.001). No significant correlations were found between age, femoral bone morphology, BMI and HOOS, and the appearance of relevant radiolucencies.

## 1. Introduction

The direct anterior approach (DAA) is thought to be a less invasive surgical approach for total hip arthroplasty (THA) [1], since this muscle-sparing approach requires shorter incisions and less soft-tissue dissection [2]. It also reduces the risk of dislocations and is associated with less postoperative pain, earlier recovery, and a lower rate of surgical complications [3]. Drawbacks include a steep learning curve for the surgeon and difficulties when using it on obese patients since with this approach more of the soft tissue release is required [3]. One of the most common complications in DAA is the increased rate of undersized femoral stems due to insufficient exposure of the femur leading to early subsidence of the femoral stem. Another complication is the associated soft tissue damage and intraoperative femoral fractures WHILE trying to improve proximal femur exposure [4].

In order to prevent these complications, some authors recommend using collared cementless stems that have shown lower complication rates in comparison with collarless stems in THA with DAA [5].

The aim of this work was to evaluate the five-year clinical and radiological outcomes in DAA THA using a cementless collared femoral short-stem. We hypothesized that the design of this implant would lead to less subsidence and a better clinical outcome when compared to the results in the literature of other short-stem implants using the DAA approach.

## 2. Materials and Methods

In this non-controlled retrospective single-center and single-surgeon cohort study, all patients aged 18 years and older and operated between 2014-2016 with a THA in Brig Hospital in Switzerland were consecutively included. Data were retrospectively reviewed for all patients who underwent THA with a DAA. Patients with revision arthroplasties or femoral neck fractures including tumor-related pathological fractures were excluded. Clinical and radiological follow-up was performed at three months, 12 months, and five years. 

All patients were operated by the same surgeon (AO) using the DAA on a traction table (RotexTable^®^, CONDOR^®^ MedTec GmbH, Salzkotten, Germany) with a specialized retractor system (CONDOR^®^ MedTec GmbH, Salzkotten, Germany). In all cases, a collared cementless triple tapered hydroxyapatite-coated femoral stem (AMIStem H Collared^®^, Medacta International, Castel San Pietro, Switzerland) in combination with a cementless cup (Versafitcup CC Trio^®^, Medacta International, Castel San Pietro, Switzerland) were implanted. Ceramic-on-ceramic bearings (MectaCer Biolox^®^ delta ceramic, Medacta International, Castel San Pietro, Switzerland) were used due to their long-term survivorship [6] and excellent tribological properties [7].

Femur morphology was categorized into three types according to the intracortical width ratio using the Dorr classification [8]. Measurement of the proximal intracortical width was performed at the mid-level of the lesser trochanter and the measurement of the distal intracortical width was performed 10 cm below the lesser trochanter. Proximal-to-distal intracortical width ratios <0.5 were considered as type A, 0.5–0.75 as type B, and 0.751–1 as type C. All radiological measurements were done by the same examiner (KP) using conventional radiographs based on the Gruen zone classification [9] to detect and localize radiolucencies. Radiolucencies <2mm in width were classified as minimal and ≥2mm in width [10] were identified as relevant since these are potential predictors for early loosening [11,12]. The 2 mm threshold was chosen in order to provide high interobserver reliability in the detection and proper interpretation of radiolucencies [13]. The radiographs were also examined to detect any subsidence of the stem. Subsidence was defined as the difference in measurements performed on the postoperative radiograph and at the last follow-up. Each measurement was defined as the distance between the most proximal point of the greater trochanter and the most prominent point of the lateral shoulder profile of the stem body. Recalibration of the radiographs and correction of magnification was achieved based on the diameter of the implanted acetabular cup [14].

Clinical outcomes were evaluated using the hip osteoarthritis outcome score (HOOS) [15], which is a valid score [16] for the assessment patient-reported outcome measurements (PROM) consisting of five subscales: pain, symptoms, activity of daily living, sport/recreation function and hip-related quality of life. The follow-up examinations were performed by a physician; however not by the operating surgeon himself.

### 2.1. Ethics Approval

This study was performed in line with the principles of the Declaration of Helsinki. Approval of this retrospective analysis was granted by the Ethics Committee of clinical research at our institution (Ethikkommission II, University Medical Centre Mannheim, Medical Faculty Mannheim, Heidelberg University, Theodor-Kutzer-Ufer 1-3, 68167, Mannheim, Approval 2020-876R).

### 2.2. Statistical Analysis

For patients’ demographics, binary variables are presented as percentages of patients per characteristic and continuous variables as mean values with standard deviations. Normality was tested using the Shapiro-Wilk-Test. The assessment of correlations between the presence of relevant radiolucencies and other categorical variables was performed using the Chi-square test. In the case of continuous variables, the *t*-test was used for normally distributed samples. For non-normally distributed samples and when the requirements for a *t*-test were not met, the Mann-Whitney U-test was used. Pre- and postoperative values were compared with paired student *t*-test or Wilcoxon signed-rank test in case the *t*-test requirements were not met. Comparison between the several time points was performed with the use of ANOVA with repeated measures. A significance level of 5% was selected. In order to measure the survival rate of the stem, the Kaplan-Meier method was used [17]. 

## 3. Results

We were able to identify 135 THA in 124 patients, 72 males (58%) and 52 females (42%), with a mean age at the time of surgery of 67.7 ± 11.3 years (range 39–88 years) and a mean body mass index (BMI) at the time of surgery of 27.4 ± 4.4 kg/m^2^ (range 17.4–41.8 kg/m^2^). Regarding femur morphology, 29% of the patients were type A according to the Dorr classification [8], 63% were classified as type B and 8% as type C. Details of patient demographics and used implants can be found in Table 1 and Table 2 respectively.

**Figure 1:**
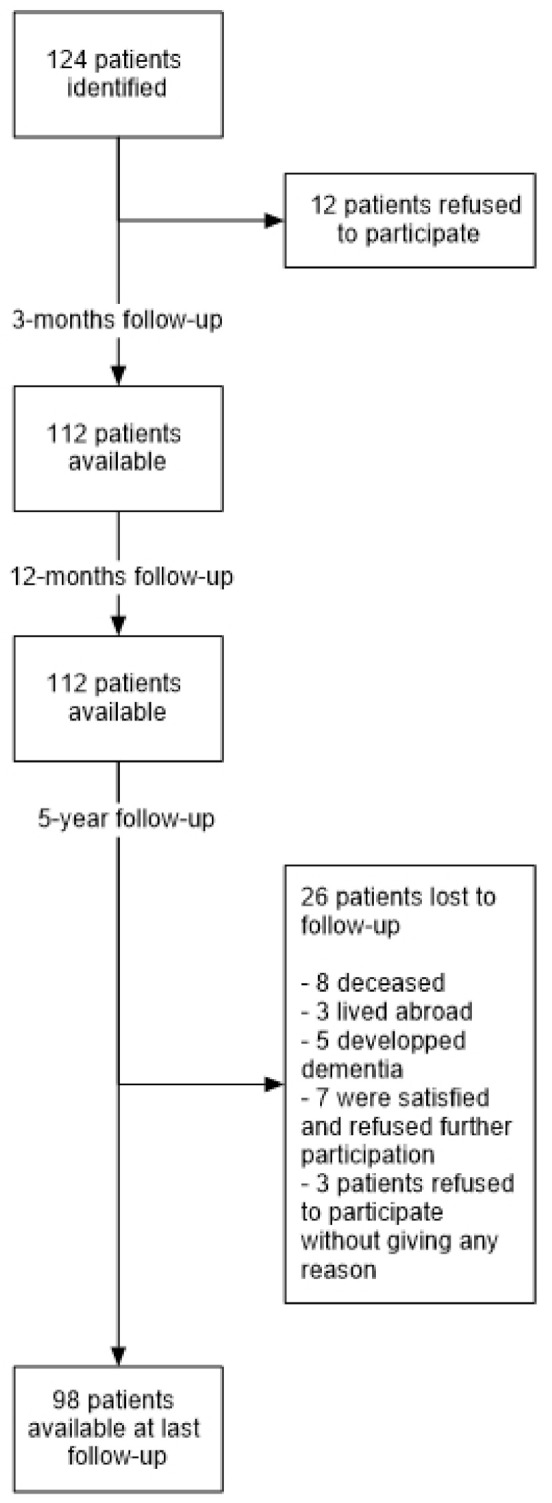
Patients available at each follow-up.

Of the identified patients, 112 patients (90.3%) with 120 hip arthroplasties were available for the three-month and the 12-month follow-up (Figure 1). Ninety-eight patients (79%) with 98 hip arthroplasties were available for all follow-up examinations including the five-year follow-up. Twenty-six patients (21%) were lost to follow-up: eight patients died (7.1%) (none of them died of a hip-related reason), three patients lived abroad, five patients developed dementia (none of them had a revision of the hip prothesis) and 10 patients refused participation in this study (seven of them were satisfied with the results and three refused to participate without giving any reason).

In one patient (0.8%), a stem revision was necessary for recurrent THA dislocations seven days postoperatively. The cause of the dislocation was an excessive retroversion of the acetabular cup. Exchange of the implanted acetabular cup with a dual mobility cup was planned. During the operation, the stem showed an unexpected relative loosening and had to be exchanged as well. In the end, a dual mobility cup and a larger stem were implanted which showed sufficient stability. The patient involved in this case was a 70-year-old obese male patient with a BMI of 37.9 Kg/m^2^. Another patient presented with a gluteal muscle insufficiency as a result of an intraoperative gluteus medius tendon tear. The insufficiency was treated non-operatively with complete recovery. A third patient presented with a periprosthetic fracture of the greater trochanter due to a fall on the hip three years after primary implantation. The fracture was treated non-operatively.

During the in-hospital stay, six patients (5%) required blood transfusions postoperatively. 

**Figure 2:**
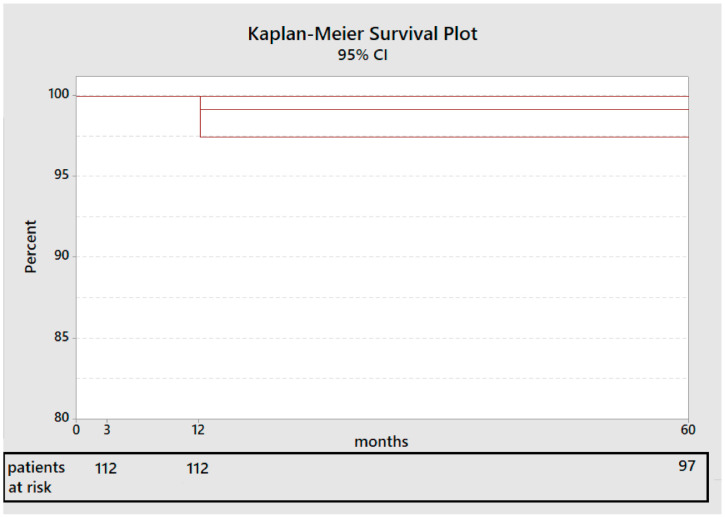
Stem survival rate (Kaplan-Meier curve. The red line represents the stem survival rate at 5 years.

No other complications were reported. The stem survival rate at five years after surgery calculated according to Kaplan-Meier Method [17] and considering revision for any reason as an endpoint was 99.15% (CI 95%: 97.7–100%) (Figure 2).

### Clinical and Radiological Outcome

At one year, 50 hips (41.7%) showed minimal radiolucencies and eight hips (6.7%) showed relevant radiolucencies of ≥2 mm. At five years, the total number of patients with radiolucencies decreased: 23 hips (23.5%) showed minimal radiolucencies and four hips (4.1%) showed relevant radiolucencies of ≥2 mm. Of these four cases, one hip showed relevant radiolucencies in zone 8, one hip in zone 2 and 8, and two hips in zone 1 and 8. 

**Figure 3:**
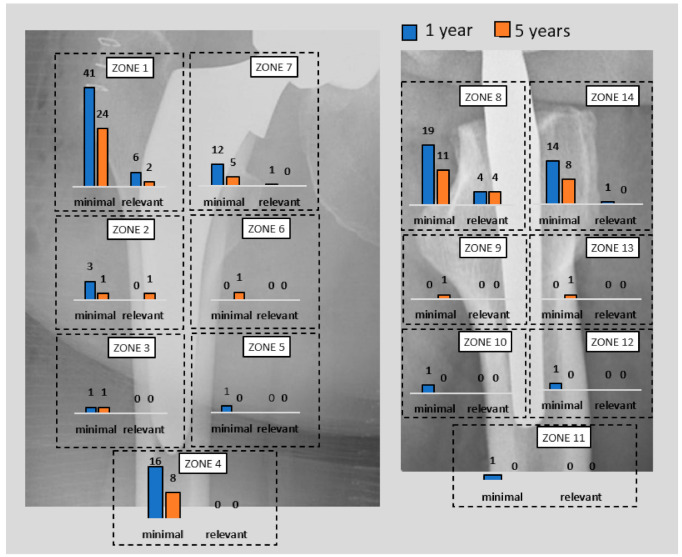
Minimal and relevant radiolucencies at 12 months and five-year follow-up according to the Gruen zones classification [13].

Of the eight relevant radiolucencies detected at the one-year follow-up, three of them could not be detected anymore at five years, two became minimal, two remained stable, and one could not be re-evaluated since the patient was not available for the last follow-up. Two minimal radiolucencies detected at one year progressed to relevant radiolucencies at five years. The two cases involved a 64-year-old male and a 54-year-old female with a BMI of 26.8 kg/m^2^ each and a Dorr type B femoral bone structure each. Details of the localization and progression of radiolucencies according to the Gruen classification [9] are presented in Figure 3.

In five THA (4.2%), subsidence of the stem was observed: in four cases (3.4%) the subsidence was <5 mm and in one patient (0.8%) >5 mm. The last patient was a 78-year-old female patient with a BMI of 26 kg/m2 and a Dorr type B femoral bone structure who showed a >5 mm subsidence of the stem, whose collar reached the lesser trochanter then stopped. In all five cases the subsidence was detected at three months. At 12 months and five years there was no progression of the subsidence. All five patients had no clinical symptoms and showed an ingrown stem at the one-year and five-year follow-ups. None of the patients with subsidence presented relevant radiolucencies.

**Figure 4:**
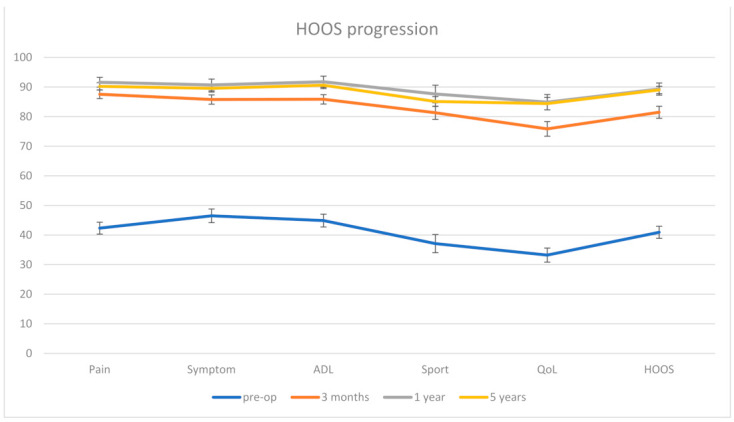
Progression of the HOOS score. The error bars represent the standard error at 95%.

80 patients completed the HOOS Questionnaire preoperatively, 91 patients at three months, 76 at 12 months and 54 at five years. The mean HOOS score improved from 40.9 ± 18.3 preoperatively to 81.5 ± 19.7 at three months, 89.3 ± 10.9 at one year and 89 ± 14 at five years (all with *p* < 0.001) (Figure 4).

None of the analyzed patient parameters (age, femoral bone morphology (Dorr classification [8]), BMI and HOOS and its subscales) showed a significant correlation with the appearance of relevant radiolucencies (*p* = 0.284–0.952). The results are shown in Table 3.

## 4. Discussion

Aim of this study was to report the radiological and clinical outcome including complication rates of THA using a collared cementless short-stem implant (AMIStem H Collared^®^, Medacta International, Castel San Pietro, Switzerland). The results of the current study confirm our hypothesis that the analyzed implant offers satisfactory results especially regarding the presence and distribution of periprosthetic radiolucencies as well as subsidence.

### 4.1. Revision Rates

The revision rate at five years in the current study was 1.2%, which is lower than other cementless short-stem prostheses and other collared stems used for THA (Table 4).

Since the surgeon has a major effect on the revision rates in THA, the single-surgeon feature of the current study may have played a role in the reduction of that rate, which may restrict the generalizability of the observed results [18]. In addition, a follow-up period of five years may be considered as limited in regard to the occurrence of complications such as loosening, subsidence, and eventual revisions.

### 4.2. Radiological Outcome

One of the potential risk factors and predictors of loosening is the presence of radiolucencies as well as their size and progression [26]. Some authors consider the mere presence of radiolucencies as a sign of loosening of the prosthesis [26]. In this study radiolucencies of ≥2 mm was identified and registered. Eight hips (6.7%) showed relevant radiolucencies at one year and four hips (4.1%) at five years. The zones affected with relevant radiolucencies at five years were the proximal Gruen zones 1, 2, and 8. This distribution of radiolucencies in the proximal part of the femur is consistent with the results of earlier studies investigating radiological outcomes of standard cementless femoral stems [27] including the Alloclassic^®^ stem (Centerpulse, Zurich, Switzerland) [28] and the Endoplus^®^ stem (PLUS Endoprothetic, Rotkreuz, Switzerland) [29] as well as outcomes of short stems such as the Optimys^®^ stem (Mathys Ltd., 2544 Bettlach, Switzerland) [30].

As mentioned earlier, radiolucencies may be related to aseptic loosening especially those ≥2 mm or those showing rapid progression [26]. However, some studies showed that the presence of these radiolucencies does not lead on the long-term (≥10 years) necessarily to aseptic loosening of the prosthesis and functional deficits [31,32]. These studies confirmed the distribution of radiolucencies in the proximal part of the femur but showed that they mostly tend not to progress [31,32] and most importantly that they do not to have an influence on the clinical outcome [27]. A possible explanation of these functionally “irrelevant” radiolucencies may be the operative technique including proximal femur preparation and prosthesis implantation [33], where slight irregularities during rasping can disrupt the cancellous bone [34]. In line with the results in the literature, a significant effect of radiolucencies on clinical outcome (HOOS score and its subscales) was not observed in the current study (*p* = 0.625). 

### 4.3. Subsidence

Distal migration of the prosthesis is another significant sign for aseptic loosening [35], which in turn is the most common cause for revision THA [36,37]. Collarless stems are thought to optimize the transmission of force and the bony support by allowing a smooth uniform loading along the whole surface of the implant. On the other hand, some authors suggest that collared stems induce more proximal loading, which reduces the distal force transmission and may negatively affect osseointegration [38,39].

**Figure 5:**
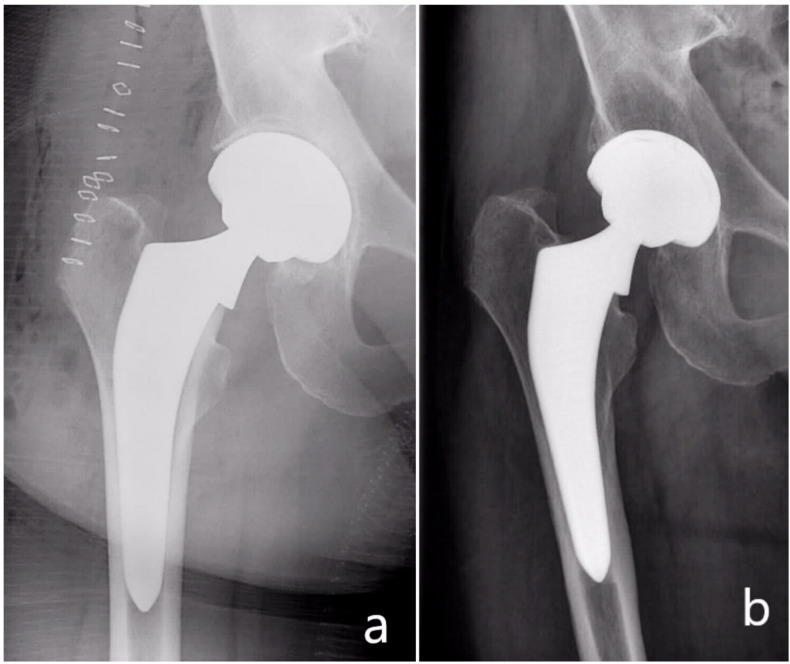
(**a**) Postoperative radiograph, (**b**) radiograph of the same patient at three months showing subsidence of the stem.

The subsidence frequency in this study was in total 4.2% (5 patients): 3.3% (4 patients) <5 mm and 0.8% (1 patient) >5 mm (Figure 5). The body mass index of these patients ranged from 26.4 to 27.2 kg/m^2^. In the postoperative radiographs, all five stems showed a neutral stem alignment and a collar to medial corticalis distance of <1 mm. The femoral neck-shaft angle in all five patients was in the normal range and varied from 129 to 134°. No fractures were detected. On the other hand, four of these five patients presented with a Dorr type B femur and one patient with a Dorr type C femur. Four of them were females. The mean age of the five patients was also 75 years higher than the total mean age of the whole collective (67.7 years). These factors, including poor bone quality, age, and femur geometry may have had a possible effect on the observed subsidence [40,41,42].

Further analysis of the illustrated case in Figure 5 shows that the subsidence occurred although the collar abuts the resection plane of the femoral neck. An explanation of the subsidence occurrence here is a possible preexisting fracture or a missed intraoperative fracture. This theory is supported by the altered cortical bone distal to the lesser trochanter. In addition, the cortical thickening medial to the tip of the stem may be a consequence of a distal force transmission, which may, in turn, suggest a non-functional collar.

However, all five cases with femoral stem subsidence were detected in the first follow-up at three months and showed no progression at the 12-month and five-year examinations. All five cases were asymptomatic. This result is comparable with those of the well-established cementless Zweymüller stem of the Alloclassic-SL^®^ system (Zimmer, Winterthur, CH), where early subsidence rates of 4% (8/198, >2 mm) and 1% (2/198, >5 mm) were reported [28].

The results of the current study are even superior to similar cementless short-stem implants: in the work of Attenello et al. from 2019 [43] a cementless short-stem (Tribute^®^, Ortho Development; Draper, UT, USA) for THA in DAA was investigated with a rate of early relevant subsidence (>5 mm) of 1,6% (4/247). Ulivi et al. [44] investigated also a short-stem (Tri-Lock BPS^®^, DePuy Synthes, Warsaw, IN, USA) implanted with the posterolateral approach and reported a subsidence rate of 4,4% (7/163). However, in this last work, the observed subsidence cases were <3 mm.

The results of Garavaglia et al. [45] are also interesting; they evaluated the outcome of the collarless version of the same implant investigated in the current study. They examined subsidence of >2 mm and noted considerably higher rates of up to 12.9%. In their study and in the current study, the prosthesis was implanted using the DAA. 

These results support the theory that the cause of the lower subsidence rate in this study may have been the collared design of the used stem and the collar-to-corticalis contact [46] that is thought to improve primary stability and consequently osseointegration by enhancing axial, varus, and rotational stability at the implant-bone interface [47]. In fact, one the most challenging issues in the implantation of the investigated stem in DAA was finding the balance between the correct size of the stem allowing press-fit stability and preventing undersizing on one hand and ensuring an optimal collar-to-corticalis contact which is essential for a functional collar on the other hand. In this context, the preoperative planning and defining the femur osteotomy line are vital to provide an optimal press-fit fixation of the stem with the correct stem size and ensure collar contact with the resection plane of the femoral neck. In an experimental work on 24 cadaveric femurs, Whiteside et al. [48] reported less subsidence and more load to failure in collared stems in comparison to collarless designs. Another factor is the length of the used stem; in DAA the challenging and sometimes insufficient femoral exposure may lead to the formation of a gap in the anterior metaphysis due to deviations in the trajectory while broaching, which in turn may eventually lead to instability and subsidence [49]. The short design of the stem can help to avoid this since the metaphyseal press-fitting prevents a diaphyseal engagement [43].

### 4.4. Periprosthetic Fracture

Femoral exposure in DAA is known to be very challenging even for skilled surgeons. Nevertheless, in the current study, there were no intraoperative periprosthetic fractures observed. In previous studies, the rates of periprosthetic femur fractures using femoral short stem prosthesis in THA with DAA were lower in comparison to those of standard-length stems [50]. Lee et al. [51] reported an incidence of periprosthetic femur fractures of 2.3% in a systemic review from 2015 including 11.810 THA using the DAA. The good results in this present study may be due to the short design of the stem that facilitates the implantation and consequently sparing the extensive femur exposure usually needed. 

### 4.5. Clinical Outcome

The main focus of the present work was on the radiological outcome since radiological signs are considered to be the best predictors of the survival of the prosthesis [52]. However, in order to assess patient satisfaction, patient-reported outcome measurements were performed.

The clinical evaluation showed a marked improvement of the patients´ postoperative HOOS scores in contrast to their preoperative status. However, comparing the clinical scores after 12 months and five years revealed a plateauing or even a decrease of some of the subscales of the HOOS score 5 years after surgery, such as the “Sport” subscore that showed a marked increase from 37.1 preoperatively to 81.3 and 87.7 after 3 and 12 months respectively, but then declined to 85.1 after 5 years. These results are in line with those observed by Garavaglia et al. [45], who analyzed the outcome of the non-collared model of the same implant examined in the current study in 698 hips using the DAA and reported Harris hip scores (HHS) of 50.9 preoperatively, 92.2 at 2 years and 90.4 at 5 years, Western Ontario McMaster Universities (WOMAC) scores of 41.6 preoperatively, 83.3 at 2 years and 80.5 at 5 years and Short-Form health survey (SF-12) physical component score of 34.5 preoperatively, 45.3 at 2 years and 43.7 at 5 years. Another possible explanation for the observed reduction in some subscales of the used scores may be the aging process of the patients during follow-up.

### 4.6. Limitations

One of the limitations of this study is the low level of evidence which is due to its retrospective design.

Although the sample size and the follow-up period is limited, it is considered to be high in comparison to other single-center and single-surgeon studies as well as to other studies involving short-stem prostheses.

Another limitation of the current study is the relatively low number of patients who completed the HOOS Questionnaire at the last follow-up. This may be considered a selection bias, since the patients that did not fill out the questionnaire may have had unsatisfactory results that were not considered in the analysis of the final clinical outcome. In addition, the HOOS score shows relatively limited reliability, structural validity, and sensitivity to change [53]. Regarding the questionnaire completion rates (80 patients preoperatively versus 91 patients at 3 months), there was no clear explanation for the increasing compliance.

A third limitation is that the clinical outcome in similar studies in the literature was not always comparable with the clinical results of the presenting work since the used evaluation scores and instruments were not always consistent and comparable. 

Furthermore, despite setting the threshold to 2 mm, a limitation of the presenting study is the relatively limited intra-observer reliability in the assessment of radiolucencies [10]. Special attention was paid to the standardization of the performed radiographs, however, a certain grade of imprecision caused by the residual rotational variance of the femur in the projections may have occurred. 

Lastly, revision rates are only one aspect of the end outcome in THA. Collared stems may be notably more difficult to revision endofemorally as the collar hinders insertion of chisels along the stem [54], leaving the transfemoral approach as the only alternative. This may lead to a stricter indication for revision and consequently create a bias in the revision rates in favor of collared stems.

## 5. Conclusions

Due to its design collared short-stem prosthesis examined in the current study provides good clinical results and shows low rates of periprosthetic radiolucencies and subsidence as well as lower revision rates in comparison to standard stems and similar other short stems.

## Figures and Tables

**Table 1:** Patient demographics (N = 124 patients, N = 135 hips).

**Sex *n* (%)**	**Males**	72 (58%)
	**Females**	52 (42%)
**Dorr Classification [8] *n* (%)**	**Type A**	39 (29%)
	**Type B**	85 (63%)
	**Type C**	11 (8%)
**Age at Time of Surgery (mean ± SD (range))**		67.7 ± 11.3 years (range 39–88 years)
BMI * at Time of Surgery (mean ± SD (range))		27.4 ± 4.4 kg/m^2^ (range 17.4–41.8 kg/m^2^)

* Body mass index.

**Table 2:** Implant details.

Implant Details		Number of Hips n (%)
AMIStem^®^ size	0–2 3–5 6–7	46 (34%) 79 (59%) 10 (7%)
Head diameter (mm)	28 32 36	46 (34%) 81 (60%) 8 (6%)
Neck length	S * M ** L ***	59 (44%) 59 (44%) 17 (12%)

* S—small; ** M—medium; *** L—large.

**Table 3:** Correlations between relevant radiolucencies and patient parameters.

Parameter	Relevant Radiolucencies		*p*-Value
	Present	Absent	
Age (mean ± SD)	63.7 ± 6.1 years	67.5 ± 11.4 years	0.329
Dorr classification			0.771
Type A	75%	68%	
Type B	25%	32%	
BMI (mean ± SD)	31 ± 5.3 kg/m^2^	27.5 ± 3.9 kg/m^2^	0.284
HOOS total (mean ± SD)	92.5 ± 7.1%	88.9 ± 14.3%	0.625
Pain	96.3 ± 5.3%	90 ± 12.3%	0.371
Symptoms	90 ± 7.1%	89.5 ± 14.8%	0.944
ADL *	95.6 ± 6.2%	90.4 ± 14%	0.477
Sport/recreation	84.4 ± 13.3%	85.2 ± 20.9%	0.952
QoL **	90.7 ± 4.5%	84.1 ± 18.4%	0.257

* Activity of daily living ** Hip related quality of life.

**Table 4:** Revision rates of other hip prosthesis stems.

Study	Prosthesis Stem	Stem Type	Approach	Revision Rates (%)	Follow-Up (Months)
Hagel et al. [19]	Mayo^®^ prosthesis (Zimmer Inc., Warsaw, IN, USA)	Collarless	Anterior	2	83.6
Falez et al. [20]			Anterior	2	56.4
Goebel et al. [21]			Posterior	10	81
Morrey et al. [22]			Anterolateral	9	78
Hallan et al. [23]	Profile^®^ (DePuy, Warsaw, IN, USA)	Collarless	Lateral	12	144
	Profile^®^ hydroxyapatite- (HA-) coated stem (DePuy, Warsaw, IN, USA)	Collarless	Lateral	4	144
Heaven et al. [24]	Corail^®^ AMT collared (DePuy, Warsaw, IN, USA)	Collared	Anterior	2.5	24
			Lateral	2.4	24
Chitnis et al. [25]	ACTIS^®^ total hip system collared (DePuy, Warsaw, IN, USA)	Collared	Anterior	1.08	36

## Data Availability

The data presented in this study are available on request from the corresponding author.
